# Ubiquinol Supplementation Improves Gender-Dependent Cerebral Vasoreactivity and Ameliorates Chronic Inflammation and Endothelial Dysfunction in Patients with Mild Cognitive Impairment

**DOI:** 10.3390/antiox10020143

**Published:** 2021-01-20

**Authors:** Sonia García-Carpintero, Javier Domínguez-Bértalo, Cristina Pedrero-Prieto, Javier Frontiñán-Rubio, Mariano Amo-Salas, Mario Durán-Prado, Eloy García-Pérez, Julia Vaamonde, Francisco J. Alcain

**Affiliations:** 1 Department of Medical Sciences, Faculty of Medicine, University of Castilla-La Mancha, 13071 Ciudad Real, Spain; Sonia.Garcia@uclm.es (S.G.-C.); CristinaM.Pedrero@uclm.es (C.P.-P.); Javier.Frontinan@uclm.es (J.F.-R.); 2 Oxidative Stress and Neurodegeneration Group, Regional Centre for Biomedical Research, University of Castilla-La Mancha, 13071 Ciudad Real, Spain; 3 Neurology Department, Virgen de Altagracia Hospital—Manzanares, SESCAM, 13002 Manzanares, Spain; javier.dominguez.bertalo@gmail.com; 4 Department of Mathematics, Faculty of Medicine, University of Castilla-La Mancha, 13071 Ciudad Real, Spain; Mariano.Amo@uclm.es; 5 Neurology Department, General University Hospital—Ciudad Real, SESCAM, 13005 Ciudad Real, Spain; eloygarciaperez@gmail.com

**Keywords:** coenzyme Q10, breath holding index, LPS, endothelial necrosis, mild cognitive impairment

## Abstract

Ubiquinol can protect endothelial cells from multiple mechanisms that cause endothelial damage and vascular dysfunction, thus contributing to dementia. A total of 69 participants diagnosed with mild cognitive impairment (MCI) received either 200 mg/day ubiquinol (Ub) or placebo for 1 year. Cognitive assessment of patients was performed at baseline and after 1 year of follow-up. Patients’ cerebral vasoreactivity was examined using transcranial Doppler sonography, and levels of Ub and lipopolysaccharide (LPS) in plasma samples were quantified. Cell viability and necrotic cell death were determined using the microvascular endothelial cell line bEnd3. Coenzyme Q10 (CoQ) levels increased in patients supplemented for 1 year with ubiquinol versus baseline and the placebo group, although higher levels were observed in male patients. The higher cCoQ concentration in male patients improved cerebral vasoreactivity CRV and reduced inflammation, although the effect of Ub supplementation on neurological improvement was negligible in this study. Furthermore, plasma from Ub-supplemented patients improved the viability of endothelial cells, although only in T2DM and hypertensive patients. This suggests that ubiquinol supplementation could be recommended to reach a concentration of 5 μg/mL in plasma in MCI patients as a complement to conventional treatment.

## 1. Introduction

Although the causes of sporadic Alzheimer’s disease (AD) are unknown, both genetic and environmental factors play important roles. Lifestyles that increase the risk of cardiovascular disease, traumatic brain injury, and oxidative stress all have been linked to AD [1,2,3]. Patients with mild cognitive impairment (MCI) have an impairment affecting one or more higher cognitive functions―often memory loss―while still maintaining functional independence and skills in their daily living. It often represents a prodromal stage in the transition between the cognitive deterioration associated with normal aging and the development of dementia symptoms, including Alzheimer’s-type dementia. It is therefore a risk marker for the subsequent development of dementia [4]. Some difficulty in its identification lies in the clinical assessment itself, as it is currently considered a heterogeneous syndrome. For this reason, a detailed neuropsychological assessment is essential to providing an accurate diagnosis. In the dementia phase, functionality is affected, and so the use of scales to assess functional impairment is becoming more common―since it is a predictor of poorer clinical evolution and higher mortality. 

Moreover, it is now widely accepted that chronic inflammation plays an important role in the onset and progression of AD [5]. Epidemiological and experimental data indicate that vascular dysfunction might contribute to dementia and the development and progression of AD, currently the most prevalent neurodegenerative disorder [2,6]. Hypertension or type 2 diabetes mellitus (T2DM) have both been found to increase the risk of cerebrovascular disease. Elevated blood pressure in middle age increases the risk of late-life dementia [7,8]. Although the association between T2DM and AD is largely independent of hypertension, it is linked to endothelial dysfunction―with T2DM being an accepted risk factor for cerebral small-vessel disease, through mechanisms involving an increase in oxidative stress in endothelial cells [2,9,10,11]. Besides hypertension and hyperglycemia, the presence of amyloid-β (Aβ) and lipopolysaccharide (LPS) can also cause endothelial dysfunction [10,11,12,13]. 

Aβ-peptide, one of the defining neuropathological features of AD, also induces oxidative stress in endothelial cells [14]. Aβ-peptide diminishes resting cerebral blood flow (rCBF) in animal models of AD [15] and reduced CBF, marking the transition from normal cognition into MCI prior to AD [16,17,18,19,20]. Besides, numerous studies have suggested that alterations in microvascular adaptability, represented by a reduction in cerebral vasoreactivity (CVR), are associated with neurodegeneration [6,21]. CBF has therefore been suggested as a biomarker of preclinical AD, since changes in brain perfusion are present long before the onset of clinical symptoms [22]. CVR can be assessed by transcranial Doppler sonography (TCD). Indeed, TCD evaluation of hypercapnia-induced CVR is considered the simplest, most inexpensive noninvasive method for assessing cerebral hemodynamics, providing a good measurement of cerebral blood flow [23,24]. 

Ubiquinol-10 (Ub), the reduced form of coenzyme Q10 (CoQ), is an antioxidant acting as an electron carrier in the mitochondrial respiratory chain. It improves endothelial function in patients with ischemic left ventricular systolic dysfunction [25], reduces inflammatory markers in patients with coronary artery disease [26], and decreases the release of LPS-induced inflammatory mediators from primary human umbilical vein endothelial cells (HUVECs) [27]. CoQ supplementation also protects endothelial cells against damage from hyperglycemia, angiotensin II or Aβ-peptide [10,12,14]. Besides, a number of trials have provided clinical evidence demonstrating that CoQ supplementation benefits endothelial function in type 2 diabetic patients and those with high blood pressure [28].

The aim of this study was (1) to analyze the effects of Ub supplementation (200 mg/day) for a 1-year period on CVR in patients diagnosed with MCI, calculated with the Breath-Holding Index (BHI); and (2) how this supplementation could be related to a reduction of chronic inflammation―measured through the presence of LPS in the patients’ plasma―and the protection of endothelial cells. For this purpose, brain microvascular endothelial cells (Bend3) incubated with plasma from patients receiving Ub or placebo were used, together with an evaluation of the potential clinical effects on these patients during the follow-up period. 

## 2. Materials and Methods

### 2.1. Participants

The study was approved by the ethics committee of the authors’ hospital and all patients provided written informed consent to their participation. The study was conducted on a group of 69 patients diagnosed with mild cognitive impairment (MCI). Patient recruitment was undertaken within the Neurology Department of the General University Hospital—Ciudad Real. MCI diagnosis was based on Petersen’s revised criteria [29] and followed several neuropsychological tests, outlined below. Each enrolled patient underwent a complete clinical history collection and blood sample analysis. The study design was a randomized, double-blind, placebo-controlled observational analytical study. The participants were randomized to receive two different treatments for a 1-year period―33 participants being supplemented with ubiquinol (Ub) and 36 with placebo. The supplements were administered as one 200 mg capsule every 24 h after breakfast. Both Ub and placebo capsules were specially produced by the same company (Kaneka Corporation) and were identical in weight and visually. All patients were older than 65 years, had been diagnosed with MCI, and showed therapeutic stability regarding any other illnesses. Exclusion criteria were dementia or other structural, metabolic infections, or pharmacological agents that could interfere with the study, i.e., carotid stenosis >50% or intracranial stenosis.

Hypertension, diabetes, and dyslipidemia were defined according to international guidelines [30,31]. Clinical evaluation of patients was performed by a neurologist expert in the diagnosis of cognitive deterioration in collaboration with a neuropsychologist who made a detailed assessment, as outlined below.

### 2.2. Cognitive Evaluation 

MCI was flagged by clinical neurologists and confirmed by the neuropsychology team. MCI diagnosis required deficits in at least two scores within different cognitive domains, or deficits in at least one score within any single domain, according to Petersen’s revised criteria [29,32], and followed several neuropsychological tests. The cognitive battery of standardized neuropsychological tests included: the Trail Making Test [33], Spain–Complutense Verbal Learning Test (Test de Aprendizaje Verbal España–Complutense, TAVEC) [34], Digit Span and Verbal Abstract Reasoning Test (WAIS-III Similarities) [35], a visuospatial span test [36], animal list generation [37], Boston Naming Test [38], Token Test [39], and the Rey–Osterrieth Complex Figure test [40]. All tests were carried out by an experienced neuropsychologist. The results of the neuropsychological tests were corrected using norms suggested by the NEURONORMA Project [41,42,43,44,45] and TAVEC. The Barthel Index (BI) [46] and the Lawton and Brody Scale (LBS) [47], the two international scales most widely used for measuring instrumental activities of daily living, were used to assess functional autonomy. Each patient enrolled in the study underwent a complete clinical history collection and blood sample analysis. Diagnostic assessments, including medical history and neurological examinations, were conducted by a neurologist with expertise in dementia. Finally, consensus diagnosis was established for the identification of MCI subjects. 

### 2.3. Transcranial Doppler Studies

Transcranial ultrasound examination was performed using transcranial Doppler sonography (TCD) (DWL Elektronische Systeme GmbH, Hamburg, Germany) with a 2 MHz ultrasound probe for transcranial examination and a 4 MHz linear vascular probe for extracranial vessels. All scans were performed in the same time slot, from 10:30 a.m. to 14:00 p.m.

Doppler sonography included: (a) internal carotid and middle cerebral arteries, to rule out hemodynamically significant stenosis; (b) in each artery, three flow velocities were measured (peak systolic velocity (PSV), mean flow velocity (MFV), and end diastolic velocity (EDV)), and (c) the Breath-Holding Index (BHI) was calculated following Markus and Harrison [48]. This test requires the subject to hold their breath for as long as possible after a normal inspiratory breath―rather than a deep breath, which might alter the results by inducing a Valsalva effect. The subjects were instructed to hold their breath for at least 20 s but no more than 30 s. They were then allowed to rest for 10 min before the test was repeated. On average, two attempts were made with each patient.

### 2.4. CoQ10 Quantification

Plasma CoQ was extracted in hexane and quantified according to a method described in a previous study [49]. Concentrations were calculated by integrating peak areas relative to external standards. On the basis of previous studies [50,51], a threshold for evaluating supplementation in plasma total CoQ (mean ± SEM) was established. The reference threshold above which supplementation was considered was 2–2.2 µg/mL. 

### 2.5. Lipopolysaccharide (Endotoxin-LPS) Concentration in Plasma

LPS in plasma samples was quantified using the Thermo Scientific Pierce LAL Chromogenic Endotoxin Quantitation Kit. Plasma samples were diluted 50-fold. A standard curve ranging between 0 and 1.0 endotoxin units per milliliter (EU/mL) was calculated. Reagents were prepared according to the manufacturer’s instructions. The assay was performed in a 96-well plate maintained at 37 °C in a heater block. After the assay was completed, the absorbance was measured on a spectrophotometer (BioRad iMark, Madrid, Spain) at 405 nm, the standard curve was calculated, and the LPS concentration of each sample was determined by interpolation.

### 2.6. Cell Cultures 

The mouse brain microvascular endothelial cell line bEnd3 (ATCC CRL-2299) was maintained at 37 °C and 5% CO_2_ in Dulbecco’s Modified Eagle Medium (DMEM, D5796 Sigma-Aldrich, Madrid, Spain), containing 10% fetal bovine plasma (FBS) and 1% antibiotic/antimycotic. The medium was refreshed every third day. All cells used in this study were up to the 18th passage. The bEnd3 cells were seeded in 96-well plates with 100 μL/well of culture media at a density of 2 × 104 cells/cm^2^ and incubated for 72 h in triplicate wells with plasma from different MCI patients (Ub or placebo). After each treatment, cells were incubated with 10 μg/mL EtBr and 1 μM Calcein-AM. Viable (green) and necrotic (red) cells were determined using Cytation 5 (BioTek, Winooski, VT, USA). A total of four images/well were taken at a 4× magnification and at least 5000 cells/well were analyzed with ImageJ. For viability and necrosis, the results are expressed as percentage vs. total cells. 

### 2.7. Statistical Analysis

GraphPad Prism v.8 software (GraphPad Inc., San Diego, CA, USA) was used for statistical analysis. A paired Student t test and one-way ANOVA were used to evaluate differences between baseline condition (T0) and 1-year follow-up (T1). Differences between groups were analyzed by unpaired Student t test. A chi-squared test was used to explore associations between two categorical variables. A Bonferroni post-hoc test was used for multiple comparisons. A Pearson correlation test was used to explore the correlation between variables. Data were represented as mean ± SEM. Differences were considered statistically significant at *p* < 0.05.

## 3. Results

### 3.1. Participants’ Cognitive Evaluation

A total of 79 MCI patients were initially enrolled in this study. From this cohort, 10 subjects were removed at a later date: 4 patients dropped out of the study voluntarily, 3 patients died before the study was completed, and 3 patients could not continue the study due to deteriorations in their health. The final number of patients included in the study was 69. The clinical characteristics of the patients at baseline are reported in Table 1. By a small margin, over half of the participants in this study were female (56.5%). The average age was 72.2 ± 5.7 years, with no significant differences between Ub and placebo groups. They were overweight, with an average BMI of 24.58 ± 5.1. A total of 29% of the patients presented with diabetes, 71% with hypertension, and 43.8% with dyslipidemia. There was no evidence of impairment in the patients’ functionality at baseline. A total of 57.5% of the patients were smokers. Baseline demographics and prevalence of hypertension, diabetes, smoking habits, dyslipidemia, BMI, Barthel Index, and Lawton and Brody scale were not statistically different between Ub and placebo groups at the beginning of the study.

### 3.2. Clinical Effects of Ubiquinol

All neuropsychological tests performed are summarized in Table 2**.** There were no significant differences in the results of the neuropsychological tests carried out between the beginning of study and after 1 year of follow-up (Table 2). At the 12-month follow-up period, based on the results of the neuropsychological evaluation, 25 patients in the Ub group had stable cognitive performance (75.8%) and seven showed a transition from MCI to dementia (21.2%), with only one patient showing a normal cognitive condition (3%). In the placebo group, 22 patients (61.1%) had stable cognitive performance, 10 (27.8%) showed a transition from MCI to dementia, and 4 patients (11.1%) had a normal cognitive condition. In line with these results, we observed a significant difference in intra-group clinical evolution (Ub group and placebo) in the Lawton and Brody scale. The patients who had progressed from MCI to dementia had lower LBS scores than MCI and normal patients in both groups (*p* ≤ 0.001), when compared T0 versus T1, but they did not show significant differences between Ub and placebo groups at any time. No patient experienced any side effects of oral ubiquinol supplementation (Table 3). 

### 3.3. Oral Supplementation with Ub Increased CoQ Concentration in Plasma

**Figure 1:**
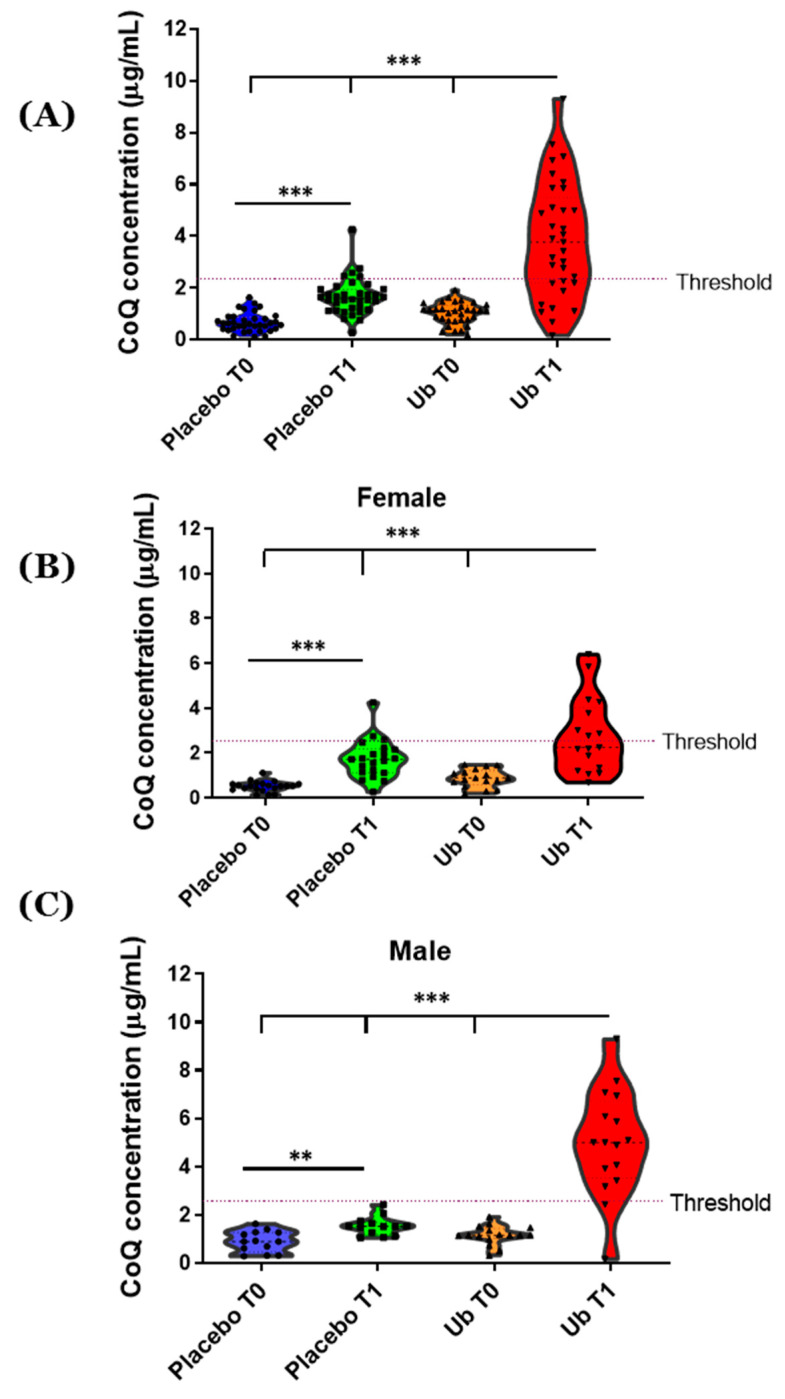
**Plasma levels of Coenzyme Q10.** Plasma levels of CoQ was evaluated by HPLC from plasma obtained from 69 patients at starting point (T0) and after 1-year supplementation (T1) with ubiquinol. (**A**) CoQ concentration in plasma from all patients enrolled and (**B**,**C**) split by sex, female and male, respectively. Values are represented in violin plots where mean and all points are indicated. Supplementation was considered for CoQ concentrations >2.1 µg/mL. Statistically significant differences were tested by Student t test, where ** and *** indicate *p* < 0.01 and *p* < 0.001, respectively.

CoQ levels at T0 were also determined and no significant differences were observed between the patients assigned to the placebo (0.730 ± 0.385 μg/mL) and the Ub groups (0.855 ± 0.400 μg/mL) (Figure 1). However, there were significant differences in CoQ plasma levels when analyzed by gender (1.040 ± 0.432 μg/mL for males vs. 0.687 ± 0.374 μg/mL for females, *p* < 0.001). Mean CoQ levels increased in the Ub group in T1 vs. baseline (Figure 1A, *p* < 0.001). Gender-dependent differences were found. Specifically, male patients showed higher CoQ levels in plasma (4.990 ± 2.204 μg/mL vs. 2.77 ± 1.677 μg/mL, *p* < 0.0027) than females after 1-year supplementation with Ub (Figure 1B,C). In the placebo group, the CoQ level was higher in T1 than in T0 (Figure 1B,C, *p* < 0.001).

### 3.4. Gender-Dependent Effects in CVR

**Figure 2:**
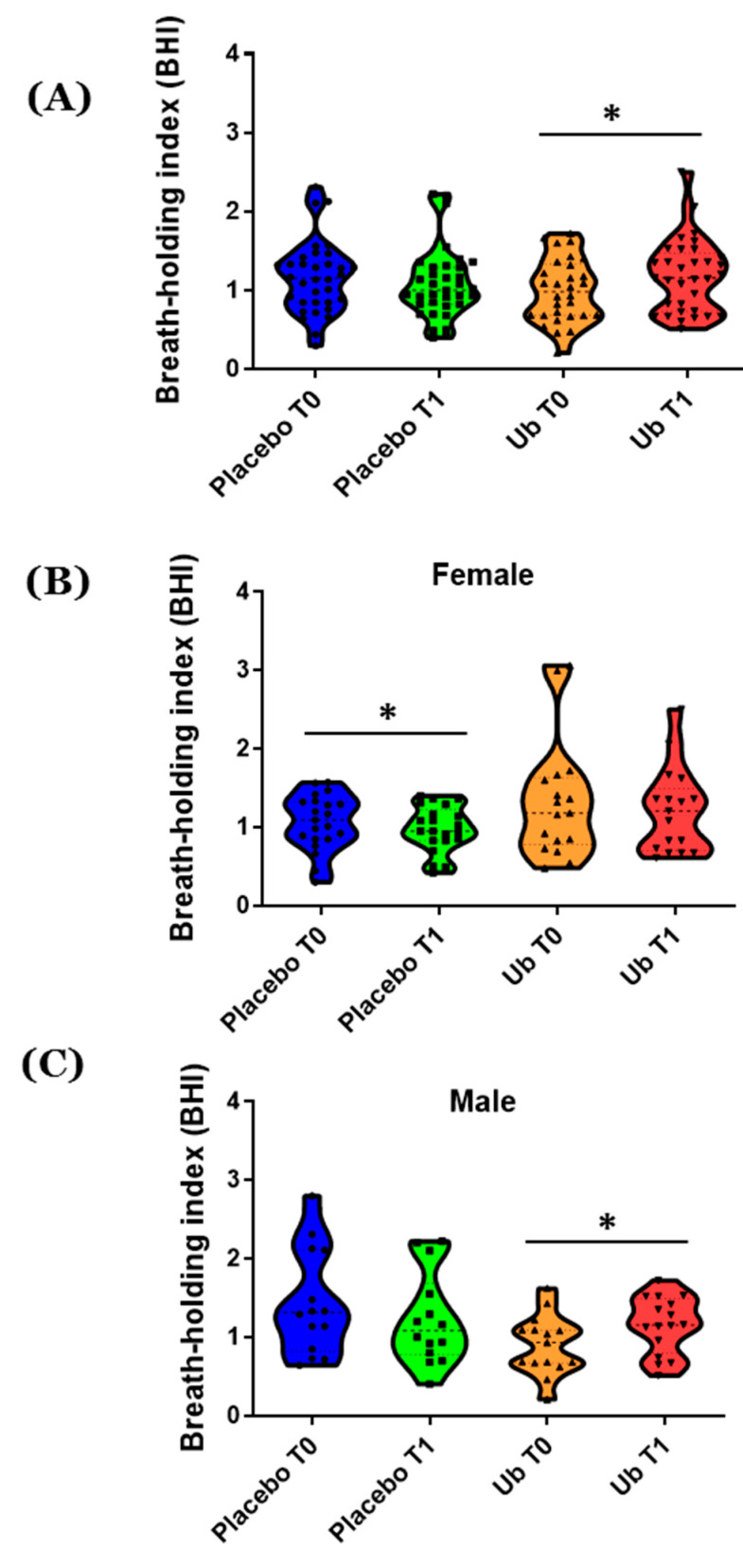
Evaluation of breath-holding index evaluation. Mild cognitive impairment (MCI) was evaluated in 69 patients at starting point (T0) and after 1-year supplementation (T1) with ubiquinol. (**A**) Total patients who participated in the study. (**B**) Female. (**C**) Male. Values are represented in violin plots where mean and all points are indicated. Statistically significant differences were tested by Student t test, where * *p* < 0.05.

BHI was measured at T0, immediately after MCI diagnosis, and at T1 after 1-year Ub supplementation. There were no significant differences in BHI values between Ub and placebo groups (Figure 2A). Additionally, males and females presented similar BHI at T0 in the UB group (1.05 ± 0.690 for males and 1.32 ± 0.740 for females). After 1 year of supplementation with Ub there were no significant differences in BHI between males and females (1.15 ± 0.362 for males and 1.208 ± 0.537 for females). However, BHI was significantly higher in the Ub group at T1 vs. T0, while in the placebo group there were no significant differences (Figure 2A, *p* < 0.05). Gender-dependent differences in total BHI levels were found both in the placebo and Ub groups. Female patients in the placebo group showed a decreased BHI at T1 vs. T0, indicating a reduction in CVR (Figure 2B, *p* < 0.05). At the same time, BHI increased significantly in male patients in the Ub group at T1 vs. T0, a clear indicator of improved CVR (Figure 2C, *p* < 0.05). 

### 3.5. Gender-Dependent Effects in Inflammation

**Figure 3:**
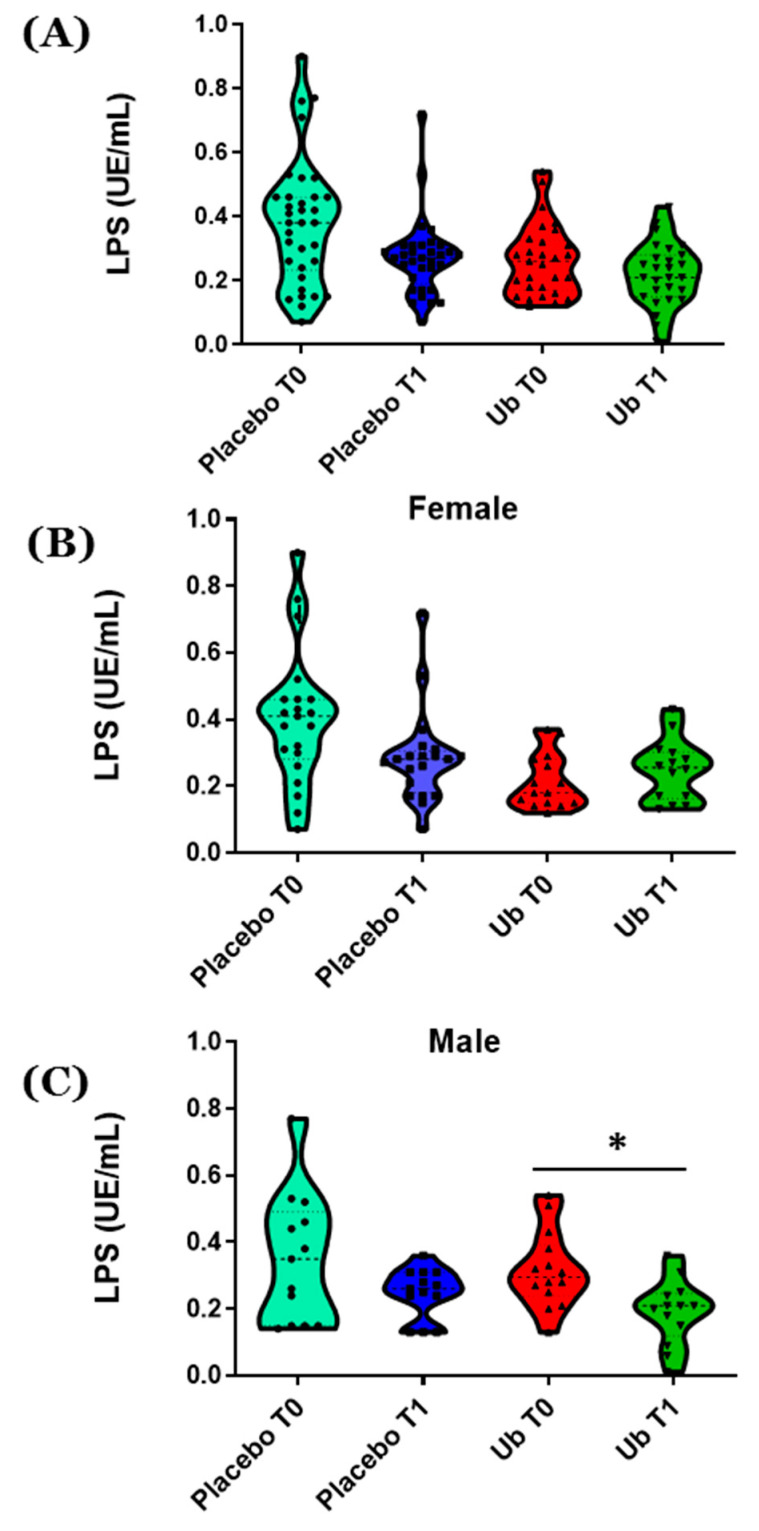
Total lipopolysaccharide (LPS) in plasma. LPS level was quantified in 69 patients at starting point (T0) and after 1-year supplementation (T1) with ubiquinol. (**A**) Total patients who participated in the study. (**B**) Female. (**C**) Male. Values, expressed as UE/mL of plasma, are represented in violin plots where mean and all points are indicated. Statistically significant differences were tested by Student t test, where * *p* < 0.05.

All patients enrolled in this study also had their LPS levels in plasma quantified in all patients enrolled in the study. At a general level, there were no differences in LPS levels neither between time points nor between placebo and Ub groups (Figure 3A). Both males and females exhibited similar levels of LPS at T0 (0.332 ± 0.154 UE/mL in males and 0.324 ± 0.187 UE/mL in females). Although after supplementation for 1 year with Ub there were no significant differences in LPS levels between males and females (0.190 ± 0.096 UE/mL for males and 0.248 ± 0.091 UE/mL for females), a decrease in LPS levels in male patients in the Ub group was found in T1 compared to T0 (Figure 3C, *p* < 0.05). This reduction in LPS levels was not found in female patients (Figure 3B). 

### 3.6. Gender-Dependent Effects on Necrotic Death Within in Vitro Brain Microvascular Endothelial Cells

Once the relationship between CoQ concentrations in plasma, CVR, and LPS levels had been analyzed, the ability of plasma to induce traumatic cell death―necrosis―in brain microvascular endothelial cells was tested. 

**Figure 4:**
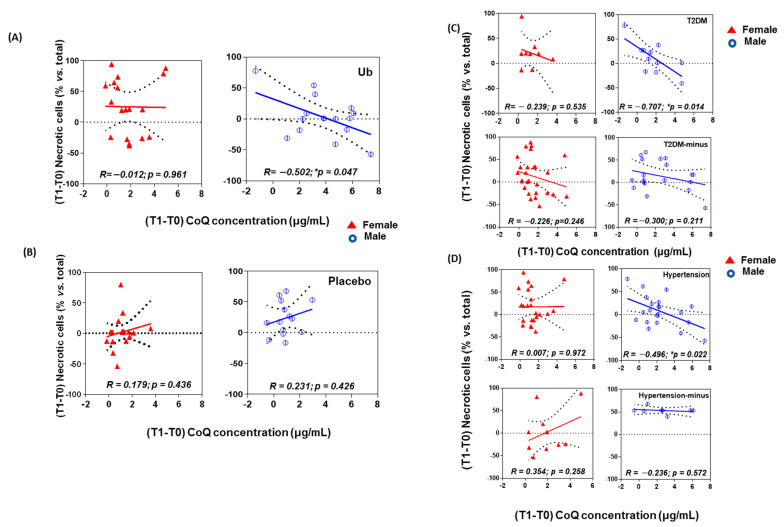
Correlation between Coenzyme Q10 concentration in plasma and necrotic cell death. Increment in CoQ concentration and in necrosis in T1-T0 are represented in X and Y axes, respectively. (**A**) Patients are stratified for Ub intake. (**B**) patients are stratified for Placebo intake. (**C**) Patients are stratified for type 2 diabetes mellitus. (**D**) Patients are stratified for hyperthension. Changes in variables were calculated as 1-year follow-up values minus baseline values.

A Pearson correlation analysis between CoQ plasma levels and in vitro endothelial cell death revealed no correlation between these parameters when considering all patients (not shown). When filtering the results within the Ub group by gender, there still was no correlation for female patients (Figure 4A, left, R = −0.012, *p* = 0.961). However, the male group revealed a negative correlation between increasing CoQ concentrations (T1–T0) and necrotic cell death (Figure 4A, right, R = −0.502, *p* = 0.047). No correlations were observed in the placebo group, neither for female patients nor males (Figure 4B).

When patients were filtered by gender and T2DM or hypertension, data from Ub and placebo groups were fused for a more potent statistical analysis due to the small sample size (Figure 4C,D). In this setting, a significant negative relationship was found between CoQ concentration (T1–T0) and necrosis-induced cell death in male patients with T2DM (Figure 4C, R = −0.707, *p* = 0.014) or hypertension (Figure 4D, R = −0.496, *p* = 0.022). No effects were observed among female patients (Figure 4C,D, R = −0.239, *p* = 0.535; R = 0.007, *p* = 0.972). 

## 4. Discussion

The mean CoQ concentration in the plasma of our patients is within the expected range for this age group, according to previously reported reference values in the literature [52], although in our case, the intraindividual differences measurements after 1 year were much higher in the placebo groups. Those patients who were randomly assigned to the placebo group presented CoQ levels in the lowest concentration range (0.664 ± 0.36 μg/mL) at T0. Furthermore, 10 patients assigned to the placebo group presented CoQ levels lower than the values reported in the literature, which can explain the significant difference in the Placebo group at T0 and T1. Furthermore, it has been reported that total CoQ plasma varies significantly with ethnicities and gender and concentrations are generally higher in males when compared to females [53,54]. Ubiquinol exhibits an acceptably safe profile as a dietary supplement up to daily doses of 300 mg over a four-week period [55], showing superior bioavailability than its reduced form ubiquinone reaching CoQ plasma levels of 4.34 ± 1.97 μg/mL [56]. Although this last study did not report data on male and female patients separately, their values were similar to those found in our study, whereas a considerable variability in the absorption of CoQ among individuals may be due to CoQ′s low bioavailability [55,56]. In the present study, males reached significantly higher CoQ plasma levels than females after 1 year of supplementation. CoQ is absorbed by enterocyte cells via a process of passive facilitated diffusion, incorporated into chylomicrons and transported to the liver, where CoQ is loaded into low-density lipoprotein (LDL) cholesterol and very-low-density lipoprotein (VLDL) cholesterol particles. A much smaller amount of CoQ is packaged into high-density lipoprotein (HDL) cholesterol [57]. The gender-dependent difference observed in CoQ concentration can be explained, at least in part, by the differences in the plasma lipid profile between males and females. Females have greater HDL concentration and lower LDL and VLDL (both during fasted and fed conditions) than age-matched men [58]. Additionally, gender differences in dietary habits have been established and could affect food–drug interactions. In general, fruit and vegetable consumption is higher in females compared with males, whereas consumption of meat and fat is higher in males compared with females [59], which could improve the intestinal solubilization of CoQ. It has been postulated that some food components play important roles in the intestinal absorption of CoQ [60]. Furthermore, the most commonly used hormone replacement therapy significantly decreases serum levels of CoQ in postmenopausal women [61]. It should be noted that supplementation with 200 mg/day for 1 year for the female group is not sufficient, for all subjects exceed the threshold of 2.1 µg/mL of CoQ. In addition, the long-term safety and tolerability of 200 mg/day Ub supplementation is noticeable, with no adverse side effects found in this study. For that, taking into consideration the excellent safety and tolerability of CoQ, it would be advisable to increase the dose in women in order to reach a therapeutic range in plasma. 

A number of studies have estimated the annual rate of conversion from MCI to dementia, mainly AD, at 10–15%, although some patients with MCI not only do not deteriorate but may actually improve over time [62,63]. In our sample this progression was greater, up to 21.2% in the Ub group and 27.8% in the placebo group. Ub supplementation did not show clear neurological benefits in these patients after a year, although it is possible that 1 year is insufficient to draw clinical conclusions on the effect of its improving the viability of endothelial cells. On the other hand, the rates of reversion to normal cognitive performance can vary from 4.5% to as high as 53% [64]. In our study, 3% of patients within the Ub group reverted to a cognitive state that was within normal levels after a year, compared to 11% in the placebo group. Therefore, it seems that the effect of Ub supplementation on neurological improvement was negligible, at least in this study, although within our group of patients, the rate of evolution to dementia was lower in the Ub group. 

It has been suggested that plasma CoQ levels higher than 2.0 μg/mL have positive effects at the cardiovascular level [65]. In line with this, our results indicate that the BHI index is higher in male patients within the Ub group, which might be due to the improvement of endothelium-dependent vasodilation in cerebral arteries in response to CO_2_ levels. This improvement was, however, not observed in female patients within the Ub group even though plasma CoQ levels of 4 μg/mL were reached; this increment only prevented CVR worsening in female patients at T1 vs. T0. On the other hand, it is noteworthy that this improvement in BHI scores among male patients does not correlate with an improvement in the patients’ neurological test results.

The association between impaired cerebral microvessel functionality in AD patients due to the chronic hypercontractility of brain vessels and unfavorable evolution of cognitive function has long been known [6,21]. CVR indicates the ability of cerebral vessels to dilate or constrict, and evidence points toward nitric oxide (NO) as an important mediator of CO_2_-related vessel dilatation [66,67]. Oxidative stress caused by different sources can dampen NO availability [68], i.e., Aβ-peptide, angiotensin II, and hyperglycemia can stimulate NADPH oxidase and decrease NO levels in endothelial cells in vitro. CoQ supplementation inhibits NADPH oxidase activity, reduces reactive oxygen species (ROS) levels, and increases NO and cell viability levels [10,12,14]. In a previous study carried out in the 3xTg-AD mouse model of AD, we described for the first time that the long-term consumption of Ub reduced the number and size of Aβ plaques and hypoxic areas found in the hippocampus and entorhinal cortex at advanced stages of the disease suggesting an improvement in the brain microvasculature [49]. Evidence suggests that changes in cerebral perfusion are present long before clinical symptoms of AD are apparent, and thus restoring the functional hyperemia of cerebral microcirculation could be crucial for maintaining normal brain function [22,68,69,70]. 

On the other hand, chronic inflammation plays an important role in the onset and progression of AD [5]. Blood LPS levels in AD patients increased three-fold vs. control, and co-localized with Aβ in amyloid plaques and around vessels in AD brains [71,72], but in the present study, a decrease in LPS levels in male patients in the Ub group was found in T1 compared to T0. Furthermore, our previous results in the 3xTg-AD model also demonstrated that hippocampal chronic inflammation could be reversed by long-term Ub oral supplementation [49,73]. This could be related to the effect of CoQ in the regulation of gene expression, since the preincubation of the human monocytic cell line THP-1 with Ub reduced the secretion of some pro-inflammatory cytokines. Furthermore, Ub delayed senescence-associated secretory phenotype acquisition by HUVECs [27]. On the other hand, LPS-induced ROS production and endothelial necrosis were totally dependent on NADPH activity [74], and CoQ can prevent its activation, caused by different insults in endothelial cells [12,75]. Therefore, LPS reduction in Ub-treated patients’ plasma could be involved in BHI improvement at least among male patients, for whom a correlation was found between higher plasma CoQ levels and lower LPS levels.

Aging is the main, irreversible risk factor for AD, but also for cardiovascular disease. Aging is linked to a reduction in plasma CoQ levels [51], which also has been associated with cardiovascular diseases and T2DM [76,77]. A negative correlation between plasma CoQ level and systolic blood pressure has been reported [78], with CoQ often being diminished in patients with T2DM [36,79]. Furthermore, lower CoQ plasma levels predicted worse performance on attention tasks in HF patients which may be related to cerebral hypoperfusion [80]. Supplementation with 5 μM CoQ is an efficient way of protecting endothelial cells in vitro against damage caused by hyperglycemia and angiotensin II [10,12]. Indeed, CoQ supplementation has substantiated clinical benefits in the prevention and treatment of hypertension and T2DM [28,40,79,81,82], and recently it has been reported that the beneficial effects of CoQ in lowering the CVD risk are associated with improving endothelial health [83]. The in vitro results presented herein regarding the effects of plasma from patients receiving Ub oral supplementation for 1 year on endothelial are limited only to the analysis of cell necrosis, and they are consistent with these data. It has been reported that Aβ peptide is seriously toxic to all the endothelium (even more than for neurons) and can contribute to the pathological breakdown of the brain–blood barrier and compromised microvascular integrity seen in AD [84]. The greater the increase in the plasma CoQ concentration, the lower the number of necrotic endothelial cells observed in the cultures, although only for male patients. These results are mirrored by BHI values and LPS levels and could have direct implications in the previously discussed improvement in analytical and vascular study parameters with transcranial Doppler. However, the sample size makes it difficult for several of the cognitive parameters analyzed to reach statistical significance. 

## 5. Conclusions

Ub oral supplementation resulted in an increase in plasma CoQ levels, with higher values in male than female patients, and no side effects. The higher CoQ concentration in male patients improved cerebral vasoreactivity and reduced inflammation. Plasma from Ub-supplemented patients improved the viability of endothelial cells, although only for T2DM and hypertensive patients. However, since these are risk factors for progression from MCI to AD, we suggest that Ub might be recommended to reach a concentration of 5 μg/mL in the plasma of patients as a complement to conventional treatment. Further trials with a longer follow-up period and a wider cohort may provide more robust results on the clinical impact of this improvement. 

## Figures and Tables

**Table 1:** Participants’ Baseline Characteristics. The values represent percentages vs. total, unless indicated otherwise. The statistically significant differences between groups at baseline were tested by ANOVA and chi-square * (*p* < 0.05). Abbreviations: BMI, body mass index; CRP, C-reactive protein.

Group Characteristics	Study Population (*n* = 69)	Ubiquinol (*n* = 33)	Placebo (*n* = 36)	*p* Value Ub vs. Placebo
Age (years) (Mean ± SEM)	72.2 (±5.7)	72.3 (±5.8)	72.08(±5.6)	0.858
Female	39 (56.5%)	17 (51.5%)	22 (61.1%)	χ2 = 0.645; *p* = 0.422
Hypertension	49 (71.0%)	23 (69.7%)	26 (72.2%)	χ2 = 0.530; *p* = 0.817
Type 2 diabetes mellitus	20 (29.0%)	7 (21.2%)	13 (36.1%)	χ2 = 1.857; *p* = 0.173
Dyslipidemia	35 (43.8%)	15 (45.5%)	20 (55.6%)	χ2 = 0.703; *p* = 0.473
Smoking	40 (57.5%)	17 (51.51%)	23 (64%)	χ2 = 0.809; *p* = 0.565
BMI (kg/m^2^) (Mean ± SEM)	24.58 (±5.1)	24.87 (±5.1)	24.32 (±5.1)	0.654
CRP (Mean ± SEM)	1.10 (±1.33)	0.92 (±0.93)	1.25 (±1.62)	0.308
Barthel scale [46]	99.71 (±1.69)	100 (±0)	99.44 (±2.32)	0.174
Lawton & Brody scale [47]	7.68 (±0.67)	7.6 (±0.7)	7.75 (±0.65)	0.380

**Table 2:** Results of baseline neuropsychological assessment at T0 and T1. Values presented as mean ± SEM for each group. Statistically significant differences between groups at baseline were tested by one-way ANOVA (*p* < 0.05).

TEST	Study Population T0 (*n* = 69)	Ubiquinol T0 (*n* = 33)	Placebo T0 (*n* = 36)	*p* Value Ub vs. Placebo T0
Part A, Trail Making Test [33]	123.97 ± 73.46	119.25 ± 55.84	128.52 ± 88.09	0.664
Digit span (forward) [35] Visuospatial span (forward) [36]	4.46 ± 0.85 4.37 ± 0.76	4.48 ± 0.90 4.48 ± 0.87	4.44 ± 0.80 4.27 ± 0.65	0.845 0.267
Part B, Trail Making Test [33] Verbal abstract reasoning (WAIS-III Similarities) [35]	273.25 ± 129.12 11.5 ± 4.01	286.91 ± 116.65 11.90 ± 3.81	257.55 ± 143.57 11.13 ± 4.20	0.464 0.435
Digit span (backward) [35] Visuospatial span (backward) [36]	3.20 ± 0.87 3.69 ± 0.82	3.18 ± 0.98 3.78 ± 0.92	3.22 ± 2.13 3.61 ± 0.72	0.839 0.880
TAVEC [34] Trial 1 and 5 free recall Delayed free and cued recall (short term) Delayed free and cued recall (long term) Recognition corrects False positives Rey–Osterrieth Complex Figure. Immediate recall [40] Rey–Osterrieth Complex Figure. Delayed recall [40]	7.60 ± 2.14 5.03 ± 2.36 6.57 ± 2.94 13.96 ± 2.55 5.52 ± 4.26 10.39 ± 5.46 9.76 ± 5.35	7.54 ± 2.20 5.03 ± 2.44 6.50 ± 2.76 14.25 ± 2.47 5.31 ± 4.14 9.00 ± 5.43 8.79 ± 5.35	7.64 ± 143.57 5.03 ± 2.33 6.64 ± 3.13 13.70 ± 2.63 5.70 ± 4.41 11.45 ± 5.30 10.54 ± 5.34	0.855 0.996 0.844 0.388 0.717 0.177 0.322
Animal list generation [37] Boston Naming Test [38] Token Test [39]	11.50 ± 4.01 39.18 ± 7.79 8.25 ± 4.18	11.77 ± 3.80 39.75 ± 8.34 8.61 ± 3.05	11.13 ± 4.20 38.66 ± 7.33 7.90 ± 4.98	0.521 0.565 0.601
Rey–Osterrieth Complex Figure. Copy [40]	25.89 ± 8.89	25.03 ± 9.11	25.80 ± 8.43	0.934
**TEST**	**Study Population T1** **(*n* = 69)**	**Ubiquinol T1** **(*n* = 33)**	**Placebo T1** **(*n* = 36)**	***p* Value** **Ub vs. Placebo T1**
Part A, Trail Making Test [33]	133.42 ± 97.23	128.12 ± 71.59	138.30 ± 117.31	0.715
Digit span (forward) [35] Visuospatial span (forward) [36]	4.42 ± 0.97 4.37 ± 0.86	4.42 ± 1.03 4.50 ± 0.75	4.41 ± 0.93 4.30 ± 0.92	0.975 0.467
Part B, Trail Making Test [33] Verbal abstract reasoning (WAIS-III Similarities) [35]	267.83 ± 118.65 10.59 ± 5.03	290.69 ± 115.76 10.93 ± 4.89	241.55 ± 119.34 10.28 ± 5.20	0.179 0.600
Digit span (backward) [35] Visuospatial span (backward) [36]	3.20 ± 0.87 3.49 ± 1.00	3.30 ± 0.80 3.72 ± 0.87	3.11 ± 0.82 3.27 ± 0.82	0.332 0.064
TAVEC [34] Trial 1 and 5 free recall Delayed free and cued recall (short term) Delayed free and cued recall (long term) Recognition correct False positives Rey–Osterrieth Complex Figure. Immediate recall [40] Rey–Osterrieth Complex Figure. Delayed recall [40]	7.98 ± 3.12 5.74 ± 2.95 6.78 ± 4.01 13.13 ± 2.63 5.03 ± 4.65 10.65 ± 6.84 10.60 ± 7.20	7.64 ± 2.13 5.66 ± 3.02 7.26 ± 3.77 13.21 ± 2.76 4.41 ± 3.45 9.23 ± 6.83 9.41 ± 7.35	7.82 ± 3.07 5.82 ± 2.94 6.35 ± 4.22 13.05 ± 2.53 5.62 ± 5.56 11.80 ± 6.79 11.57 ± 7.20	0.669 0.837 0.368 0.807 0.308 0.255 0.370
Animal list generation [37] Boston Naming Test [38] Token Test [39]	10.59 ± 5.03 38.50 ± 8.88 7.62 ± 4.21	10.61 ± 4.60 39.15 ± 9.12 6.80 ± 3.48	10.28 ± 5.20 37.91 ± 8.73 8.28 ± 4.68	0.789 0.568 0.247
Rey–Osterrieth Complex Figure. Copy [40]	25.03 ± 8.42	25.50 ± 8.88	24.90 ± 8.30	0.899

**Table 3:** Clinical parameters’ evolution of patients after ubiquinol or placebo administration. Values presented are the mean ± SEM of each group. Abbreviation: MCI Mild cognitive impair. * One-way ANOVA statistical analysis *p*-value. Row with superscripts without a common letter differ significantly *p* < 0.05 between groups in the post-hoc analysis using Bonferroni’s multiple comparisons test.

Group	Ubiquinol (*n* = 33)				Placebo (*n* = 36)			
Clinical Evolution (T1)	Dementia (*n* = 7)	Stability/MCI (*n* = 25)	Normal Cognition (*n* = 1)	Statistics	Dementia (*n* = 10)	Stability/MCI (*n* = 22)	Normal Cognition (*n* = 4)	Statistics
Barthel scale	95 ± 2.98	99.09 ± 4.26	100 ± 0	0.125	95.71 ± 7.31	99.40 ± 2.19	100 ± 0	0.090
Lawton & Brody scale	4.30 ± 8.16^a^	7.68 ± 0.89 ^b^	8 ± 0 ^b^	≥0.001 *	4.14 ± 2.11 ^a^	7.28 ± 1.17 ^b^	8 ± 0 ^b^	≥0.001 *

## Data Availability

The data generated during this study are included in this article and are available on request from the corresponding author.
